# The *VEGFB* Gene Variants and the Effectiveness of Platelet-Rich Plasma Treatment of Lateral Elbow Tendinopathy: A Prospective Cohort Study with a Two-Year Follow-Up

**DOI:** 10.3390/ijms252313166

**Published:** 2024-12-07

**Authors:** Alicja Jarosz, Tomasz Nowak, Karol Szyluk, Anna Balcerzyk-Matić, Tomasz Iwanicki, Joanna Iwanicka, Marcin Kalita, Katarzyna Gawron, Wojciech Kania, Paweł Niemiec

**Affiliations:** 1 Department of Biochemistry and Medical Genetics, Faculty of Health Sciences in Katowice, Medical University of Silesia in Katowice, Medykow 18 Str., 40-752 Katowice, Poland; alicja.jarosz@sum.edu.pl (A.J.); tnowak@sum.edu.pl (T.N.); abalcerzyk@sum.edu.pl (A.B.-M.); tiwanicki@sum.edu.pl (T.I.); jiwanicka@sum.edu.pl (J.I.); 2 District Hospital of Orthopaedics and Trauma Surgery, Bytomska 62 Str., 41-940 Piekary Slaskie, Poland; kszyluk@o2.pl (K.S.); marcin.kalita1991@gmail.com (M.K.); 3 Department of Physiotherapy, Faculty of Health Sciences in Katowice, Medical University of Silesia in Katowice, Medykow 12 Str., 40-752 Katowice, Poland; 4 Department of Molecular Biology and Genetics, Faculty of Medical Sciences in Katowice, Medical University of Silesia, Medykow 18, 40-752 Katowice, Poland; kgawron@sum.edu.pl; 5 Department of Trauma and Orthopedic Surgery, Multidisciplinary Hospital in Jaworzno, Chelmonskiego 28 Str., 43-600 Jaworzno, Poland; wojtekkania@poczta.onet.pl

**Keywords:** lateral elbow tendinopathy, LET, platelet-rich plasma, PRP, single-nucleotide polymorphism, SNP, vascular endothelial growth factor B, *VEGFB*

## Abstract

Platelet-rich plasma (PRP) is an autologous preparation used to accelerate regeneration; however, this form of therapy is not always effective. Vascular endothelial growth factor B (*VEGFB*), which affects vessel survival, pathological angiogenesis, and muscle development may differentiate the risk and treatment of lateral elbow tendinopathy (LET). In this study, we analyzed the influence of *VEGFB* gene polymorphisms on the effectiveness of LET treatment with PRP. Therapeutic effectiveness was analyzed in 107 patients (132 elbows) using patient-reported outcome measures (PROMs), specifically the visual analog scale (VAS); quick version of disabilities of the arm, shoulder, and hand score (QDASH); and patient-rated tennis elbow evaluation (PRTEE), for two years (weeks 2, 4, 8, 12, 24, 52, and 104). The polymorphisms selected for the study were rs72922019, rs12366035, rs4930152, rs594942, and rs595880, being in strong linkage disequilibrium. Patients with TT (rs72922019), TT (rs12366035), AA (rs4930152), CC (rs594942), and GG (rs595880) genotypes showed better treatment effectiveness. Statistically important differences were shown for rs72922019 VAS (week 2), QDASH (weeks 0–4), and PRTEE (week 2); rs12366035 and rs4930152 VAS (week 2), QDASH (week 2), and PRTEE (weeks 2 and 4); and rs594942 and rs595880 VAS (weeks 2 and 4), QDASH (week 2), and PRTEE (weeks 2, 52, and 104). The studied polymorphisms also showed an association with blood morphological parameters, including mean platelet volume, platelet distribution width, and eosinophil levels, as well as some comorbidities (heart failure). Genotyping due to patient selection for therapy may be considered for any of the rs72922019, rs12366035, or rs4930152 polymorphisms.

## 1. Introduction

**Figure 1:**
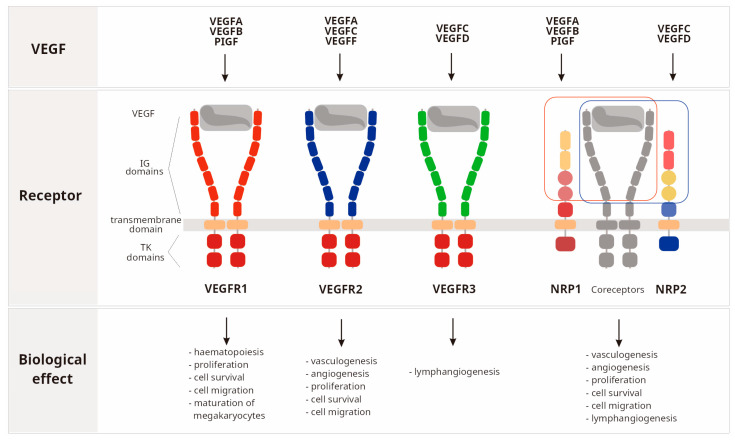
Growth factors from the VEGF family and their receptors. Selected growth factors bind to specific receptors and coreceptors. Binding to a particular receptor leads to a different biological effect [1,2,3,4,5,6]. A description is in the text. Legend: IG domain, immunoglobulin domain; TK domain, tyrosine kinase domain; VEGF, vascular endothelial growth factor; PlGF, placenta growth factor; VEGFR, vascular endothelial growth factor receptor; NRP, neuropilin.

The vascular endothelial growth factor (VEGF) family includes several members: VEGFA, VEGFB, VEGFC, VEGFD, VEGFE, VEGFF, PlGF (placenta growth factor), and EG-VEGF (endocrine gland-derived vascular endothelial growth factor) [1,2,3,4,5]. The VEGF family is a part of the platelet-derived growth factor (PDGF) superfamily that contains a cystine knot domain with eight conserved cysteine residues [3]. Signaling of growth factors from the VEGF family occurs via several receptors: fms-like tyrosine kinase 1 (VEGFR1/Flt1), human kinase insert domain receptors (VEGFR2/KDR), fms-like tyrosine kinase 4 (VEGFR3/Flt4), and neuropilins-1 and -2 (NRP1, NRP2) [1,2,3,4,5]. VEGFRs are transmembrane tyrosine kinase receptors, whereas NPRs are non-tyrosine kinase receptors that lack cytoplasmic enzyme activity [2,3]. Specific growth factors from the VEGF family bind to selected VEGFR receptors, VEGFR1: VEGFA, VEGFB, and PlGF; VEGFR2: VEGFA, VEGFC, and VEGFE; and VEGFR3: VEGFC and VEGFD [1]. Moreover, VEGFA, VEGFB, and PlGF bind to NRP1, whereas VEGFC as well as VEGFD bind to NRP2 receptors [3]. VEGF family proteins have different functions depending on the receptor they attach to. VEGF signaling through VEGFR1, VEGFR2, and NRP1 receptors regulates cell proliferation, migration, survival, and vascular permeability during vasculogenesis and angiogenesis; in turn, signaling through VEGFR3 and NRP2 is responsible for lymphangiogenesis [1,2,3] (Figure 1).

The VEGFB growth factor is encoded by the vascular endothelial growth factor B *(VEGFB)* gene (11q13.1). VEGFB-deficient mice and rats are viable without any overt phenotype, which makes it difficult to precisely determine the function of this protein. However, VEGFB appears to be a more tissue-specific vascular growth factor that may have trophic and metabolic effects [3]. VEGFB has a cardioprotective role [7], regulates myocardial contractility and metabolism, and may influence atherogenesis [8]. In addition, the administration of VEGFB stimulates neurogenesis in adult mice, while VEGFB-deficient mice were found to have impaired recovery from ischemic brain injury [3]. VEGFB treatment protects endangered neurons from apoptosis without inducing undesired blood vessel growth [9]. Overall, VEGFB appears to be important for tissue protection, whether this occurs in the context of heart failure or neuronal degeneration [3]. Moreover, VEGFB inhibits apoptosis of pericytes, smooth muscle cells, and vascular stem/progenitor cells [7,8,9]. As for the role of VEGFB in angiogenesis, it was unclear for a long time. Ultimately, studies have shown that VEGFB is not responsible for the development of blood vessels but is necessary for their survival [7,8,9]. The vascular survival effect of VEGFB is achieved by regulating the expression of multiple pro-vascular survival genes via both VEGFR1 and NPR1 signaling [7,9]. In addition, the inhibition of its expression reduces pathological angiogenesis [8,9]. Recent studies have also shown that VEGFB can inhibit angiogenesis by suppressing fibroblast growth factor 2 (FGF2) signaling through the FGF2/FGFR1 pathway [10].

Platelet-rich plasma (PRP) is an autologous preparation obtained from a patient’s blood. Therefore, many factors may influence its properties, including age [11], sex [12], growth factors’ concentration [13], and genetic variation [14,15,16,17,18]. PRP is often used in treating tendinopathies [19]; however, there are other treatment options such as surgery, non-steroidal anti-inflammatory drugs, orthotic devices, physiotherapy, or corticosteroid injections [20]. Since selecting the right therapy enables a faster and inexpensive recovery, finding factors that predispose patients to PRP therapy can facilitate the treatment.

The aim of the current study was to investigate the association between *VEGFB* gene polymorphisms and the effectiveness of PRP therapy in the treatment of lateral elbow tendinopathy (LET). VEGFB is present in whole blood [21]. Although these data indirectly indicate its presence in PRP as well, to our knowledge, there are no data on this in the literature. It is known, however, that this growth factor is strongly associated with vascular survival and pathological angiogenesis [7,8,9]. Proper angiogenesis is very important in the treatment of tendinopathy, while pathological angiogenesis may contribute to its development [14,22]. Therefore, growth factors associated with the development and survival of vessels may potentially affect the effectiveness of the treatment. Moreover, in our previous study, we showed that single-nucleotide polymorphisms (SNPs) of the *VEGFA* gene are indeed associated with the effectiveness of PRP therapy [14]; thus, we decided to examine the influence of other proteins from the same family. This publication is part of a larger study analyzing the influence of genetic factors on the effect of PRP therapy. So far, five genes encoding growth factors from the PDGF superfamily, and their receptors, have been studied [14,15,16,17,18]. This study is based on the analysis of the association between five different *VEGFB* gene polymorphisms (rs72922019 (C>T), rs12366035 (C>T), rs4930152 (G>A), rs594942 (T>C), and rs595880 (T>G)) and patient-reported outcome measure (PROM) values in the following weeks after PRP administration. Due to the lack of data on the impact of *VEGFB* gene SNPs on tendinopathy or PRP therapy, the polymorphisms for the study were selected mainly based on their appropriate frequency in the studied population.

## 2. Results

### 2.1. VEGFB Gene Polymorphisms

**Figure 2:**
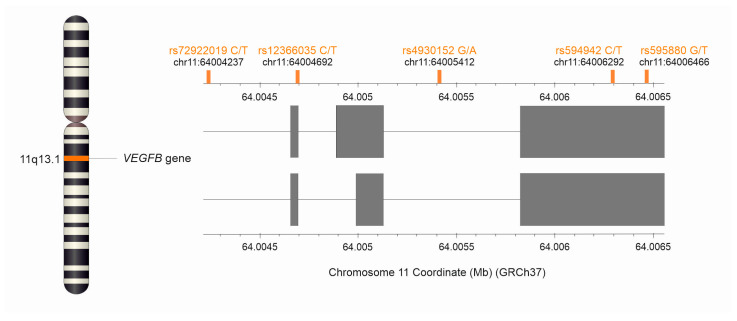
Locations of the studied *VEGFB* polymorphisms. The figure was created using data from the LDmatrix tool [24].

Five polymorphisms of the *VEGFB* gene were selected for the study: rs72922019, rs12366035, rs4930152, rs594942, and rs595880. The rs72922019 and rs4930152 polymorphisms are intron variants, and rs594942 and rs595880 are 3-prime UTR variants, whereas rs12366035 is a synonymous variant [23]. Figure 2 shows the *VEGFB* gene with the location of the studied SNPs. Alleles and genotypes’ frequencies for studied polymorphisms are shown in Table 1.

**Figure 3:**
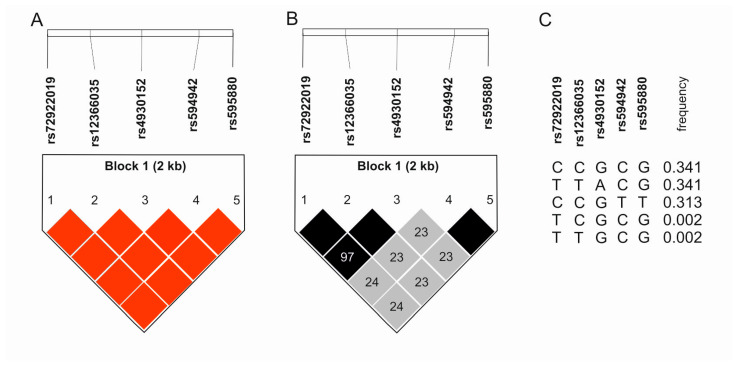
Linkage analyses between studied polymorphisms. (**A**) Haplotype block with D’ value. (**B**) Haplotype block with R^2^ value. (**C**) Haplotypes and their frequency. Colors highlight the degree of linkage disequilibrium. The darker the color, the greater the D’ or R2 values.

Based on the data from the National Center for Biotechnology Information database, studied SNPs are in strong linkage disequilibrium. High values for both D’ and R^2^ (both equal to 1.0) occur for two blocks: rs72922019, rs12366035, and rs4930152; and rs594942 and rs595880. Comparing all five SNPs, it can be seen that they are still strongly linked to each other, although there are clear differences in the D’ and R^2^ values (D’ equals 1.0, R^2^ equals around 0.227) [23]. In turn, analyses of our study group give similar results, clearly indicating the formation of one large haplotype block for all the studied polymorphisms. The results are shown in Figure 3.

### 2.2. Association Between the Studied Polymorphisms and PROM Values

**Figure 4:**
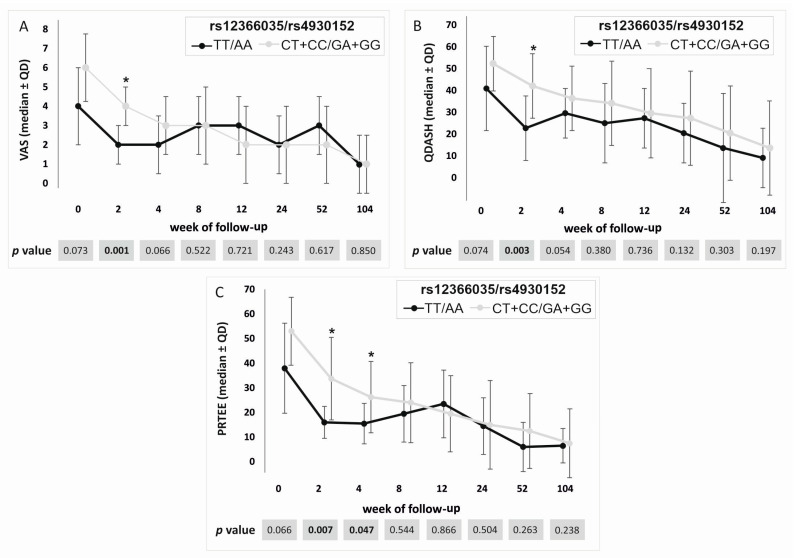
Medians (±QD) of PROM values for carriers of different rs12366035 and rs4930152 genotypes. Results for (**A**) VAS, (**B**) QDASH, and (**C**) PRTEE. Legend: QD, quartile deviation; PROM, patient-reported outcome measure; VAS, visual analog scale; QDASH, quick version of disabilities of the arm, shoulder, and hand score; PRTEE, patient-rated tennis elbow evaluation; *, significant difference (*p* < 0.050).

Although the studied SNPs showed such strong linkage disequilibrium, there were still slight differences in the results. In the case of the rs72922019, statistically important differences were shown for visual analog scale (VAS) in week 2, quick version of disabilities of the arm, shoulder, and hand score (QDASH) in weeks 0-4, and patient-rated tennis elbow evaluation (PRTEE) in week 2 in the dominant/recessive model. Therapy was more effective for carriers of the TT genotype. Unfortunately, the difference at baseline (week 0), i.e., before the start of therapy, indicates that the results for this polymorphism are not very reliable in the case of QDASH. The results for the rs12366035 and rs4930152 SNPs were nearly identical; therefore, they are presented together in Figure 4. Carriers of the TT (rs12366035) and the AA (rs4930152) genotypes were characterized by lower values of VAS (week 2), QDASH (week 2), and PRTEE (weeks 2 and 4). Differences for the same follow-up weeks were also observed for the rs594942 and rs595880 polymorphisms. Values of VAS (weeks 2 and 4), QDASH (week 2), and PRTEE (weeks 2, 52, and 104) were lower for the CC (rs594942) and the GG (rs595880) homozygotes. After Hochberg correction, the *p* value for multiple tests was assessed as 0.007. With such *p* values, the results for the rs72922019, rs12366035, and rs4930152 for VAS and QDASH at week 2 as well as rs4930152 for PRTEE at week 2 remained statistically significant. Detailed results are shown in Supplementary Materials (Tables S1–S5).

In the additive model, identical results characterized two separate groups of SNPs; one included the rs72922019, rs12366035, and rs4930152, and the other rs594942 and rs595880. In the case of the first group, homozygotes TT (rs72922019), TT (rs12366035), and AA (rs4930152) had lower values of VAS, QDASH, and PRTEE in the second week of follow-up compared to carriers of other genotypes. Meanwhile, in the case of the rs594942 and rs595880, carriers of the CC (rs594942) and the GG (rs595880) genotypes had lower values of VAS in weeks 2 and 4 (Table S6). Hochberg correction for the additive model determined a *p* value of 0.012, maintaining statistically significant results for the rs72922019, rs12366035, and rs4930152 for VAS at week 2.

### 2.3. Association Between the Studied Polymorphisms and Blood Count Parameters

Studied polymorphisms showed an association with blood count parameters. The rs72922019, rs12366035, and rs4930152 were associated with mean platelet volume (MPV), platelet distribution width (PDW), mean corpuscular volume (MCV), mean corpuscular hemoglobin concentration (MCH), eosinophil (EOS), and basophil (BASO) values. Meanwhile, the rs594942 and rs595880 showed an association with EOS, lymphocytes (LYMs), monocytes (MONOs), and red blood cell distribution width (RDW). Interestingly, genotypes associated with better therapy effectiveness (TT rs72922019, TT rs12366035, and AA rs4930152) were characterized by higher values of MPV and PDW as well as lower values of MCV, MCH, and EOS in the dominant/recessive model (Table 2). The results for BASO showed no differences in median values (the statistical test examines the distribution, not the median value, although there are differences in the mean values: 0.034 for CC and 0.029 for CT+TT of rs72922019). In the additive model, statistically important differences occurred only for the EOS level (Table S7). For the CC (rs594942) and GG (rs595880) genotypes that showed greater therapy efficacy, higher MONO levels as well as lower EOS, LYM, and RDW levels were observed in the dominant/recessive model (Table 2). Differences between MONO, EOS, and RDW levels were also visible in the additive model (Table S7). After Hochberg correction, the results for EOS (for all analyzed SNPs) in the dominant/recessive model (*p* = 0.007), and RDW (for the rs594942 and rs595880) in the additive model (*p* = 0.001), remained statistically significant.

### 2.4. Association Between the Studied Polymorphisms and Comorbidities

In order to examine whether confounding factors influenced the study results, we analyzed the association of studied SNPs with the comorbidities. The rs72922019 and rs4930152 were associated with autoimmune diseases and hypertension, while the rs12366035 only with autoimmune diseases. In turn, the rs594942 and rs595880 showed an association with heart failure. After Hochberg correction, the *p* value was determined at the level of 0.00038, classifying as statistically significant only the association between the rs594942, rs595880, and heart failure. TT genotypes (both for rs594942 and rs595880) were associated with a higher incidence of heart failure. These genotypes were also characterized by lower treatment efficacy (detailed results in Table S8). After removing patients with the above-mentioned comorbidities from the study group, the results for the studied SNPs showed the same trend. There were slight differences in the results, but the same genotypes were still associated with greater or lesser treatment effectiveness.

## 3. Discussion

The current study showed that all studied *VEGFB* gene SNPs were associated with the efficacy of lateral elbow tendinopathy treatment with platelet-rich plasma. However, after applying corrections for multiple testing, statistically significant associations were maintained only for the rs72922019, rs12366035, and rs4930152. Moreover, the association between the genotype and therapy was mainly visible in the early stages of treatment (around 2-4 weeks after injection). This may indicate that *VEGFB* is involved in the initial stages of regeneration following PRP administration.

**Figure 5:**
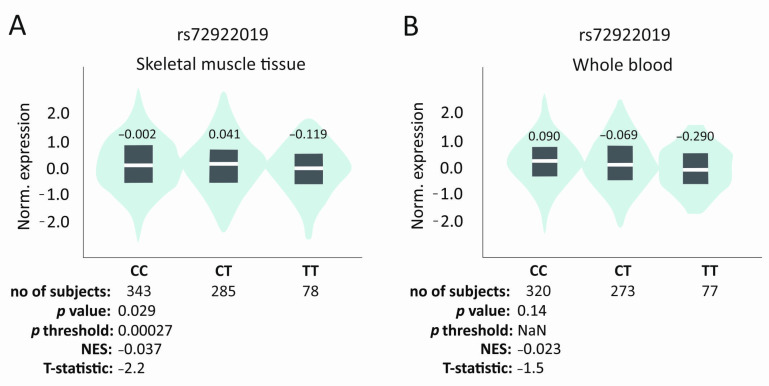
In silico analysis of rs72922019 *VEGFB* gene polymorphism influence on expression level. Results for (**A**) skeletal muscle tissue and (**B**) whole blood. Based on GTEx Portal [24].

In silico analyses showed a trend according to which TT homozygotes of the rs72922019 polymorphism have the lowest level of *VEGFB* gene expression (in skeletal muscle tissues and whole blood), although these results were not statistically significant (Figure 5) [25]. The exact mechanism by which rs72922019, as an intronic polymorphism, may affect *VEGFB* expression is not known. However, it should be noted that in general, SNPs may influence gene expression, mRNA conformation, or the subcellular localization of mRNA and/or proteins [26].

Interestingly, the TT genotype of the rs72922019 was also associated with higher therapy effectiveness in our study. Studies in mice have shown that loss of VEGFB/VEGFR1 signaling is associated with skeletal muscle development [27]. Moreover, patients with LET showed reduced wrist extensor strength compared to healthy individuals [28]. Therefore, reduced *VEGFB* activity, which translates into muscle growth, may lower the risk of developing tendinopathy. Lower activity of the *VEGFB* gene can also be beneficial in treating tendinopathy since its inhibition reduces pathological angiogenesis [8,9]. Pathological angiogenesis is one of the main processes responsible for the progression of tendinopathy [29]. Studies have shown that higher expression levels of angiogenesis-related factors such as VEGF can be harmful to tendon healing [30]. What is more, VEGFB is also responsible for blood vessel survival [7,8,9]. These two functions of the VEGFB may explain our results, showing association with therapy only in the early stages. Lower *VEGFB* expression and reduced pathological angiogenesis may be beneficial in early stages of regeneration but normal vessel survival is also important for proper healing [14,22]. The determination of the exact *VEGFB* role in tendinopathy treatment requires more detailed research. The influence of studied SNPs on *VEGFB* gene expression in different tissues, especially tendons and ligaments, also requires a comprehensive analysis.

In addition, the examined SNPs were associated with hematological parameters. Although the association of the *VEGFB* gene polymorphisms with various blood morphological elements was observed, platelet parameters are particularly important when examining PRP. Unfortunately, there is a lack of information in the literature on the effect of VEGFB on platelet formation. In this context (as well as in many others), VEGFA is a more thoroughly studied protein. However, studies indicate that megakaryocyte maturation is associated with signaling via the VEGFR1 receptor [6], whose ligand is also VEGFB [1,2,3,4,5]. Determining whether VEGFB is also involved in this process requires research. Genotypes associated with higher PRP efficacy (rs72922019, rs12366035, and rs4930152 polymorphisms) were associated with higher MPV and PDW values. This is consistent with our previous studies indicating that platelet volume (and therefore platelet activity) affects PRP effectiveness [17,18]. These results did not remain statistically significant after the Hochberg correction, but still show some trends. Only the results indicating that the genotypes associated with better therapy efficacy are also associated with lower EOS levels (for rs72922019, rs12366035, and rs4930152) and lower RDW levels (for rs594942 and rs595880) remained statistically significant. The results related to the EOS values are particularly interesting. Our results are consistent with a genome-wide association study, which showed that rs4930152 is associated with an increased amount and percentage of eosinophils [31], further suggesting that *VEGFB* may be involved in hemopoiesis. Our previous study analyzing the effect of blood morphological parameters on PRP activity showed that a higher level of eosinophils had a positive effect on the effectiveness of treatment [32]. Eosinophils have a significant effect on wound healing, remodeling [33], and tissue regeneration [34]. It should be emphasized, however, that eosinophils are also associated with angiogenesis. They have been shown to promote angiogenesis as a result of hypoxia [35]. Most tendons are poorly vascularized and respond to hypoxia by secreting angiogenic factors (such as VEGF), which is associated with the pathogenesis of tendinopathy [22]. Based on this information, it can be assumed that maintaining a certain balance is important for proper regeneration. According to our results, the median EOS values for genotypes associated with better therapy progress were 0.12-0.13 [10*^9^/L], and for those associated with worse progress, 0.19-0.21 [10*^9^/L] (Table 2). For comparison, in the cited study of blood morphology parameters and PRP, patients with better therapy progress had EOS equal to 0.17 [10*^9^/L] [32]. Perhaps most important for the proper progress of therapy is an adequate level of eosinophils (not too low and not too high). Of course, these considerations require a more detailed examination.

The studied SNPs also showed an association with comorbidities, although after applying the correction for multiple testing, only the association between the rs594942, rs595880, and heart failure remained statistically significant. Despite this, comorbidities had no significant impact on the study results. Removal of patients with these comorbidities from the study group did not disturb the study outcome. The influence of rs594942 and rs595880 polymorphisms on *VEGFB* activity may be related to translation regulation. The 3-prime UTR regions are responsible for mRNA stability, its subcellular localization, and translation control [36]. Polymorphisms present in this region may modify these processes. Moreover, according to the Ensembl database, rs595880 is a sequence variant located within a regulatory region (enhancer) [37]. Association between the *VEGFB* SNPs and heart failure seems probable due to VEGFB’s role in cardioprotection [3,7]. However, studies have shown that diseases related to the circulatory system can also affect the risk of tendinopathy. For example, patients with atherosclerotic cardiovascular disease may have an increased risk of tendon rupture and tendinopathy due to an increased incidence of comorbidities and the use of medications associated with tendinopathy [38]. In addition, musculoskeletal disorders (such as carpal tunnel syndrome, biceps tendon rupture, and low back pain) are considered a warning sign for the risk of transthyretin-associated amyloid cardiomyopathy [39]. The genotypes of the rs594942 and rs595880 polymorphisms, which were associated with better treatment efficacy (CC and GG, respectively), were also associated with a lower incidence of heart failure in the study group, which may be related to a lower risk of tendinopathy development for these patients.

The current study has several limitations. These include the relatively small study group, no restrictions on additional therapy forms after PRP injection, and the lack of a control group. Although the patients received varying volumes of PRP due to differences in their hematocrit levels/hydration status at the time of injection (different PRP volumes were obtained after the centrifugation of the same blood volume), we believe that this did not significantly affect the outcomes. We did not include a control group because the current study aimed to analyze the influence of genetic factors on the effectiveness of PRP, not its overall effectiveness. Therefore, the groups compared in this study are carriers of different genotypes. The inclusion of patients who used additional forms of therapy in the study was primarily due to ethical issues. In our opinion, it is unethical to prohibit patients with ineffective PRP therapy from accessing other forms of treatment for two years. Furthermore, to limit the influence of the small group on the results, we focused primarily on quantitative analyses, which are less sensitive to statistical analyses. Nevertheless, additional research is necessary to confirm the obtained results.

## 4. Materials and Methods

### 4.1. Study Design

This study followed the STROBE and MIBO guidelines, with the research protocol approved by the Ethics Committee of the Medical University of Silesia in Katowice, Poland (KNW/0022/KB1/24/I/17). The methodology complied with the principles of the 1975 Helsinki Declaration and its subsequent amendments, ensuring ethical conduct. All participants provided written informed consent.

The study employed the same effectiveness measures, follow-up schedule, patient selection criteria, PRP injection procedures, and blood analyses as our previous research [14,15,16,17,18]. The patient cohort included individuals diagnosed with lateral elbow tendinopathy who received PRP treatment. Genetic analyses of selected SNPs of the *VEGFB* gene and blood morphology were performed.

### 4.2. Measures of Effectiveness and Follow-Up

Therapy progress was monitored over a 2-year period at intervals of 2, 4, 8, 12, 24, 52, and 104 weeks post-injection using PROMs, specifically VAS, QDASH, and PRTEE. The VAS ranged from 0 (no pain) to 10 (maximum pain), while QDASH and PRTEE scores spanned from 0 (no pain or disability) to 100 (maximum pain and disability). We used culturally adapted Polish versions of the QDASH [40] and PRTEE [41] questionnaires. Results were compared to each patient’s baseline clinical condition at the time of injection (week 0).

### 4.3. Patient Selection and Characteristics

**Figure 6:**
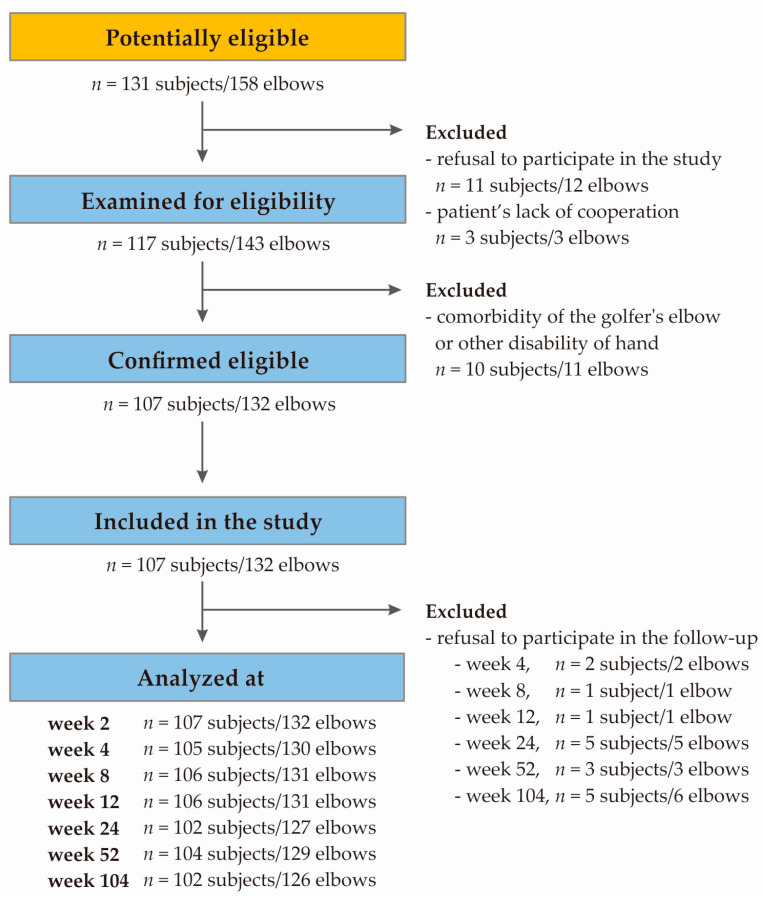
Flow chart presenting selection of studied group.

The selection of patients took place between November 2018 and November 2019, with data collection continuing until November 2020. A flow diagram of the study selection process is provided below (Figure 6). The study involved 107 Polish Caucasian participants from Upper Silesia, including 65 women and 42 men, aged between 24 and 64 years (median ± QD: 46.00 ± 5.50). All were diagnosed with lateral elbow tendinopathy (with 25 cases of bilateral involvement), classified under the ICD-10 code M77.1. Importantly, age did not affect therapy effectiveness. All patients displayed characteristic LET symptoms such as pain at the origin of the common extensor, tenderness upon the palpation of the lateral epicondyle, muscle weakness, morning stiffness, a history of limb overuse or injury, and positive results for Thomson’s, Mill’s, and Cozen’s tests.

The exclusion criteria included other injuries or conditions (e.g., rheumatoid arthritis, active cancer, cervical radiculopathy), prior PRP injections, previous surgeries, steroid injections within the last 6 months, use of anti-platelet medications, or pregnancy. Importantly, the study did not implement a specific rehabilitation protocol post-injection. Additional therapies following the injections, such as steroids, nonsteroidal anti-inflammatory drug use, physiotherapy, or further PRP injections, were monitored but did not serve as exclusion factors.

The average concentration of white blood cells (WBCs) was 6.26 ± 1.16 (10^*9^/L ± QD), the platelet (PLT) count was 240.00 ± 40.50 (10^*9^/L ± QD), and the MPV was 9.10 ± 0.73 (fL ± QD). Women had higher platelet levels (261.50 ± 33.00 vs. 224.00 ± 38.75, *p* = 0.000) and plateletcrit (PCT) (2.37 ± 0.36 vs. 2.04 ± 0.33, *p* = 0.001) in whole blood compared to men. The presence of comorbidities, such as diabetes, renal failure, gout, cancer, spleen diseases, autoimmune diseases, hypertension, hypercholesterolemia, and heart failure, was also noted and did not affect the effectiveness of therapy. Common comorbidities in the group included hypertension, thyroid disorders, and gout. A summary of demographic and clinical data is presented in Table 3.

### 4.4. PRP Separation and Injection Procedure

Whole blood was collected under standardized conditions in a treatment room equipped with disposable materials. PRP was separated from fresh whole blood immediately after collection. The plasma was extracted using an Arthrex Autologous Conditioned Plasma double syringe (Arthrex GmbH, München, Germany). For each patient, 12 mL of blood was drawn using a 1.2 mm needle, mixed with 3.13% sodium citrate (MediPac^®^ GmbH, Königswinter, Germany) in a 9:1 ratio, and centrifuged under consistent conditions with a Rotofix 32A centrifuge (Andreas Hettich GmbH & Co., Tuttlingen, Germany) at 1500 rpm for 5 min. For patients with bilateral lateral elbow tendinopathy, PRP was extracted separately for each elbow. A 2.0–3.0 mL PRP injection was administered immediately into the common extensor origin using a 1.2 mm needle, guided by ultrasound with a Mindray DC-3 machine (Mindray Medical Poland Sp. z o.o., Warsaw, Poland).

Patients received autologous PRP treatment at either the VI Department of Trauma and Orthopedics in the District Hospital of Orthopedics and Trauma Surgery in Piekary Śląskie, Poland, or the Department of Orthopedic Trauma Surgery in the Multidisciplinary Hospital in Jaworzno, Poland. The same orthopedic surgeons (K.S. in Piekary Śląskie and W.K. in Jaworzno) selected, examined, and treated all patients according to an identical study protocol.

### 4.5. Genetic Analyses

Peripheral blood was collected from the patients for genetic testing. DNA was isolated from the blood using the MasterPure genomic DNA purification kit (Epicenter Technologies in Madison). DNA was then used to genotype five selected SNPs of the *VEGFB* gene, rs72922019, rs12366035, rs4930152, rs594942, and rs595880, whose minor allele frequency in the European population was above 20% [23]. Genotyping was performed using TaqMan assays (Thermo Fisher Scientific in Waltham, MA, USA) and the Real-Time PCR LightCycler^®^480 (F. Hoffmann-La Roche AG, Bazylea, Switzerland). Genotyping was repeated for 15% of the samples to confirm the repeatability of the results. The results were 100% repeatable. Genotyping was nearly 100% successful. It failed for one patient in the case of the rs1236603 polymorphism.

### 4.6. Whole-Blood Parameters

The collected peripheral blood was used to perform a complete blood count. The assessed parameters included WBC, red blood cells (RBCs), hemoglobin (HGB), hematocrit (HCT), MCV, MCH, mean corpuscular hemoglobin concentration (MCHC), RDW, PLT, MPV, PDW, PCT, neutrophils (NEUs), LYM, MONO, EOS, and BASO.

### 4.7. Statistical Analysis

Statistical analyses were performed using Statistica 13.0 software (TIBCO Software Inc., Palo Alto, CA, USA). Data distribution normality was assessed with the Shapiro–Wilk test. As the quantitative variables did not follow a normal distribution, the Mann–Whitney U test was applied for comparisons. Quantitative results were presented as the median with the interquartile range (QD). Statistical significance was defined as *p* < 0.050. The Hochberg correction was calculated for multiple comparisons to adjust the *p* values [42]. Cases with missing data were excluded from the relevant analyses. The genotype frequencies of the studied SNPs were in accordance with Hardy–Weinberg equilibrium.

Genetic analyses included an association between *VEGFB* genotypes, therapy effectiveness, and blood parameters. The association between *VEGFB* genotypes and treatment effectiveness was evaluated by analyzing raw VAS, QDASH, and PRTEE scores as well as changes from baseline values (ΔVAS, ΔQDASH, and ΔPRTEE). Genetic data were analyzed using dominant/recessive and additive inheritance models. We employed the Mann–Whitney U test in the dominant/recessive model and the Kruskal–Wallis test in the additive model. Haplotype blocks were identified with HaploView 4.2 software (Broad Institute of MIT and Harvard, Cambridge, MA, USA) [43], following the algorithm of Gabriel et al. [44]. D′ and R^2^ values were used to assess linkage disequilibrium. The χ^2^ test was used to determine the association between *VEGFB* genotypes and comorbidities, with Yates correction for subgroups of less than 10 patients. During additional analyses, patients with comorbidities (autoimmune diseases, hypertension, heart failure) were excluded from the study group in order to verify the association between the genotype and therapy efficacy without confounding factors.

## 5. Conclusions

In conclusion, in the current study, we demonstrated an association between the *VEGFB* gene polymorphisms and the treatment of lateral elbow tendinopathy with PRP. All of the studied SNPs showed an association with the effectiveness of the treatment, although the most significant results were obtained for the rs72922019, rs12366035, and rs4930152. In addition, the trend seen in the in silico analysis showed that TT rs72922019 homozygotes may have the lowest expression of the *VEGFB* gene. An association of the analyzed polymorphisms with blood morphological parameters was also demonstrated. In terms of PRP effectiveness, the results related to platelet parameters and eosinophil levels are particularly important. Finally, the rs594942 and the rs595880 polymorphisms showed a strong association with heart failure, which may be a risk factor in the case of tendinopathy. The association of the *VEGFB* with the treatment of tendinopathy seems to be mainly related to the influence of this protein on pathological angiogenesis and muscle development.

## Figures and Tables

**Table 1:** Studied *VEGFB* polymorphisms along with chromosomal location and genotypes’ frequencies.

*VEGFB* SNP	Chromosome 11 Coordinate *	Allele	*n*	%	Genotype	*n*	%	MAF %, **
rs72922019	64004237	C	114	60.00	CC	56	42.42	
		T	76	40.00	CT	58	43.94	29.3
					TT	18	13.64	
					CC+CT	114	86.34	
					TT+CT	76	57.56	
rs12366035	64004692	C	114	60.32	CC	56	42.75	
		T	75	39.68	CT	58	44.27	29.3
					TT	17	12.98	
					CC+CT	114	87.02	
					TT+CT	75	57.25	
rs4930152	64005412	G	115	60.21	GG	56	42.42	
		A	76	39.79	GA	59	44.70	29.3
					AA	17	12.88	
					GG+GA	115	87.12	
					AA+GA	76	57.56	
rs594942	64006292	C	119	63.98	CC	65	49.24	
		T	67	36.02	CT	54	40.91	35.4
					TT	13	9.85	
					CC+CT	119	90.15	
					TT+CT	67	50.76	
rs595880	64006466	G	119	63.98	GG	65	49.24	
		T	67	36.02	GT	54	40.91	35.4
					TT	13	9.85	
					GG+GT	119	90.15	
					TT+GT	67	50.76	

Legend: *VEGFB*, vascular endothelial growth factor B; SNP, single-nucleotide polymorphism; MAF, minor allele frequency. * GRCh37. q13.1 chr 11, ** 1000 Genomes, Europe.

**Table 2:** Differences in blood morphology parameters in carriers of different genotypes of *VEGFB* polymorphisms (dominant/recessive model).

SNPs	Blood Count Parameter	Genotypes				*p* Value
		Median	QD	Median	QD	
^1^ rs72922019		^1^ TT/^2^ TT/^3^ AA		^1^ CT+CC/^2^ CT+CC/^3^ GA+GG		
^2^ rs12366035	MPV [fl]	10.10	0.85	9.00	0.65	0.043
^3^ rs4930152	PDW [fl]	16.30	0.10	16.00	0.15	0.025
		^1^ CC/^2^ CC/^3^ GG		^1^ CT+TT/^2^ CT+TT/^3^ GA+AA		
	MCV [fl]	93.60	2.70	91.30	3.25	0.010
	MCH [pg]	30.90	1.05	29.90	1.00	0.012
	EOS [%]	2.70	1.50	2.00	0.60	0.047
	EOS [10*^9^/L]	0.21	0.09	0.12	0.04	0.006 *
^4^ rs594942		^4^ CC/^5^ GG		^4^ CT+TT/^5^ GT+TT		
^5^ rs595880	EOS [10*^9^/L]	0.13	0.04	0.19	0.07	0.007 *
	LYM [10*^9^/L]	1.65	0.28	1.92	0.31	0.032
	MONO [%]	5.50	1.50	4.50	1.20	0.009
		^4^ TT/^5^ TT		^4^ CT+CC/^5^ GT+GG		
	RDW [%]	12.40	0.40	11.90	0.33	0.015

Legend: SNP, single-nucleotide polymorphism; QD, quartile deviation; MPV, mean platelet volume; PDW, platelet distribution width; MCV, mean corpuscular volume; EOS, eosinophil; LYM, lymphocyte; MONO, monocyte; RDW, red blood cell distribution width. *, statistically significant after Hochberg correction (*p* = 0.007).

**Table 3:** Characteristics of the study group.

Characteristics			
General	number of subjects, n	107	–
	number of elbows, n (%)	132	(100.0)
	tennis elbow in dominant hand, n (%)	86	(65.2)
	tennis elbow in non-dominant hand, n (%)	46	(34.8)
	females, n (%)	77	(58.3)
	age, median ± QD	46.00	5.50
	BMI, median ± QD	25.65	2.00
	overweight/obesity BMI ≥ 25, n (%)	86	(65.2)
	current smokers, n (%)	22	(16.6)
	former smokers, n (%)	48	(36.4)
Comorbidities	diabetes mellitus, n (%)	4	(3.0)
	gout, n (%)	8	(6.1)
	thyroid diseases, n (%)	15	(11.4)
	hypercholesterolemia, n (%)	10	(7,58)
	hypertension, n (%)	18	(13.6)
	heart failure, n (%)	4	(3.0)

Legend: BMI, body mass index; PLT, platelet; PCT, plateletcrit; MPV, mean platelet volume; PDW, platelet distribution width; PRP, platelet-rich plasma; QD, quartile deviation.

## Data Availability

The original contributions presented in the study are included in the article/Supplementary Material, further inquiries can be directed to the corresponding author/s.

## Supplementary Materials

The following supporting information can be downloaded at: https://www.mdpi.com/article/10.3390/ijms252313166/s1.
