# Supplementary material for: The VEGFB Gene Variants and the Effectiveness of Platelet-Rich Plasma Treatment of Lateral Elbow Tendinopathy: A Prospective Cohort Study with a Two-Year Follow-Up
Source: Int J Mol Sci. 2024 Dec 7;25(23):13166. doi: 10.3390/ijms252313166 (PMC11642232; doi:10.3390/ijms252313166)
Supplement: Supplementary file 1 [file ijms-25-13166-s001.zip › ijms-3332559-supplementary.pdf]

Table S1. PROMs values in carriers of different genotypes of the rs72922019 polymorphism of the *VEGFB* gene.

PROMs values in TT homozygotes and carriers of the C allele of the rs72922019 *VEGFB* gene polymorphism.

PROMs		TT rs72922019		CT+CC rs72922019		*p* Mann-Whitney U test
	week	median	± QD	median	± QD	
VAS	0	4.00	3.00	6.00	1.50	0.145
	2	2.00	1.50	4.00	1.00	**0.004***
	4	2.00	1.50	3.00	1.50	0.093
	8	3.00	1.50	3.00	2.00	0.530
	12	2.50	1.50	2.50	2.00	0.686
	24	2.00	1.50	2.00	2.00	0.248
	52	2.00	1.50	2.00	2.25	0.572
	104	1.00	1.50	1.00	1.50	0.851
ΔVAS (vs week 0)	2	2.00	1.00	1.00	1.50	0.206
	4	2.50	2.00	2.00	1.75	0.965
	8	2.00	2.00	3.00	2.00	0.607
	12	2.50	2.00	3.00	2.00	0.427
	24	2.00	1.50	3.00	2.00	0.760
	52	2.00	1.50	4.00	2.50	0.416
	104	2.50	1.50	4.00	2.50	0.214
QDASH	0	39.77	21.60	52.27	11.37	**0.032**
	2	23.86	14.77	40.91	14.77	**0.006***
	4	29.55	11.36	36.36	14.77	**0.046**
	8	23.86	18.18	34.09	19.32	0.311
	12	27.27	13.64	29.55	20.45	0.725
	24	21.59	13.64	27.27	21.59	0.150
	52	10.23	25.00	20.45	21.59	0.231
	104	10.23	11.93	14.77	21.59	0.201
ΔQDASH (vs week 0)	2	4.32	11.36	6.81	13.64	0.797
	4	7.95	12.50	13.63	14.77	0.300
	8	11.36	18.18	15.91	18.18	0.317
	12	11.36	19.32	18.18	17.04	0.140
	24	16.82	21.59	20.45	18.18	0.648
	52	13.63	22.73	22.72	19.32	0.432
	104	25.00	22.16	29.55	22.73	0.589
PRTEE	0	39.00	18.25	53.00	13.75	0.083
	2	17.50	9.00	33.50	16.75	**0.011**
	4	16.00	8.50	26.50	14.50	0.052
	8	18.50	11.50	24.25	16.25	0.463
	12	21.75	13.75	19.75	15.50	0.955
	24	15.00	11.50	14.50	18.00	0.525
	52	6.00	10.00	12.75	15.38	0.226
	104	6.75	6.00	7.75	14.00	0.246
ΔPRTEE (vs week 0)	2	18.50	9.50	14.50	11.88	0.247
	4	23.75	10.25	21.25	14.50	0.671
	8	27.75	9.25	25.50	16.95	0.748
	12	27.50	13.50	29.50	16.00	0.390
	24	31.25	15.25	30.50	19.50	0.856
	52	29.50	18.75	33.50	18.25	0.749
	104	35.25	17.63	38.50	16.50	0.938

PROMs values in CC homozygotes and carriers of the T allele of the rs72922019 *VEGFB* gene polymorphism.

PROMs	week	CC rs72922019 median	CC rs72922019 ± QD	CT+TT rs72922019 median	CT+TT rs72922019 ± QD	*p* Mann-Whitney U test
VAS	0	6.00	1.50	6.00	1.88	0.788
	2	4.00	1.50	4.00	1.50	0.815
	4	3.00	1.50	3.00	1.50	0.873
	8	2.50	1.50	3.00	2.00	0.415
	12	2.00	1.75	3.00	1.50	0.757
	24	1.00	2.50	2.00	2.00	0.349
	52	1.00	2.50	2.00	2.00	0.923
	104	1.00	2.00	1.00	1.50	0.301
ΔVAS (vs week 0)	2	2.00	1.75	1.00	1.50	0.535
	4	2.00	1.50	2.00	2.00	0.544
	8	3.00	2.50	2.00	2.00	0.424
	12	3.00	2.00	3.00	2.00	0.751
	24	3.00	2.50	2.00	1.50	0.410
	52	3.50	3.00	3.50	2.00	0.903
	104	4.00	2.50	4.00	2.50	0.336
QDASH	0	48.86	10.16	52.27	14.20	0.914
	2	40.91	13.07	38.64	17.05	0.551
	4	38.64	14.20	34.09	14.77	0.586
	8	34.09	19.89	31.82	16.48	0.806
	12	23.86	22.73	29.55	14.20	0.757
	24	14.77	21.59	25.00	20.45	0.443
	52	22.73	26.14	17.05	21.59	0.849
	104	18.18	25.00	9.09	14.78	0.117
ΔQDASH (vs week 0)	2	4.54	15.34	6.81	12.63	0.644
	4	12.50	15.33	12.49	15.91	0.890
	8	14.77	19.94	15.91	17.05	0.858
	12	18.18	16.48	18.18	18.18	0.921
	24	23.86	20.46	20.45	18.18	0.498
	52	23.86	21.59	22.72	19.32	0.734
	104	29.54	23.87	30.68	21.02	0.240
PRTEE	0	50.75	13.00	53.25	14.88	0.753
	2	28.75	12.13	31.50	18.50	0.976
	4	24.75	14.38	24.50	13.75	0.877
	8	21.25	15.25	24.25	16.88	0.668
	12	16.00	15.38	21.50	14.50	0.537
	24	9.50	17.00	18.75	16.75	0.237
	52	12.75	13.75	11.50	14.63	0.776
	104	8.50	17.50	6.50	9.75	0.218
ΔPRTEE (vs week 0)	2	17.50	13.15	14.00	13.25	0.965
	4	21.75	14.25	20.75	12.75	0.839
	8	27.75	14.25	24.50	17.50	0.961
	12	26.75	14.73	29.00	18.25	0.786
	24	33.50	17.50	30.50	19.00	0.667
	52	35.75	15.50	32.50	18.00	0.439
	104	37.00	19.75	39.00	17.25	0.085

Legend: *VEGFB*, vascular endothelial growth factor B, QD, Quartile Deviation; VAS, Visual Analog Scale; QDASH, quick version of Disabilities of the Arm, Shoulder and Hand score; PROM, Patient-Reported Outcome Measures; PRTEE, Patient-Rated Tennis Elbow Evaluation. * - statistically significant after Hochberg correction ($p = 0.007$).

Table S2. PROMs values in carriers of different genotypes of the rs12366035 polymorphism of the *VEGFB* gene.

PROMs values in TT homozygotes and carriers of the C allele of the rs12366035 *VEGFB* gene polymorphism.

**PROMs**		**TT rs12366035**		**CT+CC rs12366035**		***p***
	week	median	± QD	median	± QD	**Mann-Whitney U test**
VAS	0	4.00	2.00	6.00	1.50	0.078
	2	2.00	1.00	4.00	1.00	**0.001***
	4	2.00	1.50	3.00	1.50	0.067
	8	3.00	1.50	3.00	2.00	0.516
	12	3.00	1.50	2.50	2.00	0.711
	24	2.00	1.50	2.00	2.00	0.241
	52	3.00	1.50	2.00	2.25	0.612
	104	1.00	1.50	1.00	1.50	0.845
ΔVAS (vs week 0)	2	2.00	1.00	1.00	1.50	0.260
	4	2.00	2.00	2.00	1.75	0.796
	8	2.00	2.00	3.00	2.00	0.443
	12	2.00	2.00	3.00	2.00	0.266
	24	2.00	1.50	3.00	2.00	0.571
	52	2.00	1.50	4.00	2.50	0.255
	104	2.00	1.50	4.00	2.50	0.121
QDASH	0	40.90	19.32	52.27	11.37	0.067
	2	22.73	14.77	40.91	14.77	**0.003***
	4	29.55	11.36	36.36	14.77	0.053
	8	25.00	18.18	34.09	19.32	0.367
	12	27.27	13.64	29.55	20.45	0.732
	24	20.45	13.64	27.27	21.59	0.137
	52	13.64	25.00	20.45	21.59	0.294
	104	9.09	13.64	14.77	21.59	0.195
ΔQDASH (vs week 0)	2	4.55	11.36	6.81	13.64	0.920
	4	11.35	12.50	13.63	14.77	0.453
	8	11.36	18.18	15.91	18.18	0.430
	12	11.36	17.04	18.18	17.04	0.237
	24	22.27	20.45	20.45	18.18	0.876
	52	15.90	22.73	22.72	19.32	0.522
	104	38.63	26.14	29.55	22.73	0.752
PRTEE	0	38.00	18.25	53.00	13.75	0.068
	2	16.00	6.50	33.50	16.75	**0.008**
	4	15.50	8.25	26.50	14.50	**0.048**
	8	19.50	11.50	24.25	16.25	0.530
	12	23.50	13.75	19.75	15.50	0.873
	24	14.50	11.50	14.50	18.00	0.502
	52	6.00	10.00	12.75	15.38	0.255
	104	6.50	7.00	7.75	14.00	0.235
ΔPRTEE (vs week 0)	2	17.50	9.50	14.50	11.88	0.275
	4	21.50	10.25	21.25	14.50	0.743
	8	24.50	9.00	25.50	16.95	0.608
	12	27.00	13.25	29.50	16.00	0.290
	24	31.00	15.25	30.50	19.50	0.745
	52	27.00	14.50	33.50	18.25	0.601
	104	33.50	21.50	38.50	16.50	0.847

PROMs values in CC homozygotes and carriers of the T allele of the rs12366035 *VEGFB* gene polymorphism.

PROMs	week	CC rs12366035		CT+TT rs12366035		*p* Mann-Whitney U test
		median	± QD	median	± QD	
VAS	0	6.00	1.50	6.00	1.75	0.719
	2	4.00	1.50	4.00	1.50	0.770
	4	3.00	1.50	3.00	1.50	0.842
	8	2.50	1.50	3.00	2.00	0.418
	12	2.00	1.75	3.00	1.50	0.747
	24	1.00	2.50	2.00	2.00	0.349
	52	1.00	2.50	2.00	2.00	0.934
	104	1.00	2.00	1.00	1.50	0.300
ΔVAS (vs week 0)	2	2.00	1.75	1.00	1.50	0.497
	4	2.00	1.50	2.00	2.00	0.488
	8	3.00	2.50	2.00	2.00	0.377
	12	3.00	2.00	2.50	2.00	0.675
	24	3.00	2.50	2.00	1.50	0.362
	52	3.50	3.00	3.25	2.00	0.987
	104	4.00	2.50	4.00	2.50	0.378
QDASH	0	48.86	10.16	52.27	13.64	0.818
	2	40.91	13.07	38.64	17.05	0.513
	4	38.64	14.20	35.23	14.77	0.611
	8	34.09	19.89	31.82	17.05	0.833
	12	23.86	22.73	29.55	14.77	0.761
	24	14.77	21.59	25.00	20.45	0.444
	52	22.73	26.14	18.18	21.59	0.885
	104	18.18	25.00	9.09	15.34	0.118
ΔQDASH (vs week 0)	2	4.54	15.34	6.81	11.50	0.552
	4	12.50	15.33	13.63	14.77	0.815
	8	14.77	19.94	15.91	17.05	0.795
	12	18.18	16.48	19.32	17.05	0.991
	24	23.86	20.46	20.45	18.11	0.564
	52	23.86	21.59	22.72	19.32	0.695
	104	29.54	23.87	31.81	20.46	0.212
PRTEE	0	50.75	13.00	53.00	15.25	0.768
	2	28.75	12.13	30.25	18.50	0.994
	4	24.75	14.38	25.00	13.75	0.872
	8	21.25	15.25	24.50	17.25	0.631
	12	16.00	15.38	21.50	14.75	0.515
	24	9.50	17.00	19.50	16.75	0.242
	52	12.75	13.75	11.50	14.75	0.755
	104	8.50	17.50	6.25	10.63	0.220
ΔPRTEE (vs week 0)	2	17.50	13.15	14.00	13.25	0.975
	4	21.75	14.25	20.50	12.75	0.863
	8	27.75	14.25	24.50	17.50	0.901
	12	26.75	14.73	28.50	18.25	0.839
	24	33.50	17.50	29.50	19.25	0.625
	52	35.75	15.50	32.25	18.00	0.481
	104	37.00	19.75	38.50	18.25	0.092

Legend: *VEGFB*, vascular endothelial growth factor B; QD, Quartile Deviation; VAS, Visual Analog Scale; QDASH, quick version of Disabilities of the Arm, Shoulder and Hand score; PROM, Patient-Reported Outcome Measures; PRTEE, Patient-Rated Tennis Elbow Evaluation. * - statistically significant after Hochberg correction (p = 0.007).

Table S3. PROMs values in carriers of different genotypes of the rs4930152 polymorphism of the *VEGFB* gene.

PROMs values in AA homozygotes and carriers of the G allele of the rs4930152 *VEGFB* gene polymorphism.

PROMs		AA rs4930152		GA+GG rs4930152		*p*
	week	median	± QD	median	± QD	Mann-Whitney U test
VAS	0	4.00	2.00	6.00	1.75	0.073
	2	2.00	1.00	4.00	1.00	**0.001***
	4	2.00	1.50	3.00	1.50	0.066
	8	3.00	1.50	3.00	2.00	0.522
	12	3.00	1.50	2.00	2.00	0.721
	24	2.00	1.50	2.00	2.00	0.243
	52	3.00	1.50	2.00	2.00	0.617
	104	1.00	1.50	1.00	1.50	0.850
ΔVAS (vs week 0)	2	2.00	1.00	1.00	1.50	0.275
	4	2.00	2.00	2.00	1.50	0.769
	8	2.00	2.00	3.00	2.00	0.416
	12	2.00	2.00	3.00	2.00	0.245
	24	2.00	1.50	3.00	2.00	0.543
	52	2.00	1.50	4.00	2.50	0.237
	104	2.00	1.50	4.00	2.50	0.112
QDASH	0	40.90	19.32	52.27	12.50	0.074
	2	22.73	14.77	42.05	14.77	**0.003***
	4	29.55	11.36	36.36	14.77	0.054
	8	25.00	18.18	34.09	19.32	0.380
	12	27.27	13.64	29.55	20.45	0.736
	24	20.45	13.64	27.27	21.59	0.132
	52	13.64	25.00	20.45	21.59	0.303
	104	9.09	13.64	13.64	21.59	0.197
ΔQDASH (vs week 0)	2	4.55	11.36	6.81	13.63	0.875
	4	11.35	12.50	13.63	14.77	0.489
	8	11.36	18.18	15.91	18.19	0.453
	12	11.36	17.04	18.18	17.04	0.254
	24	22.27	20.45	20.45	18.19	0.913
	52	15.90	22.73	22.72	19.32	0.532
	104	38.63	26.14	29.55	22.73	0.774
PRTEE	0	38.00	18.25	53.00	13.75	0.066
	2	16.00	6.50	33.75	16.75	**0.007***
	4	15.50	8.25	26.25	14.50	**0.047**
	8	19.50	11.50	24.00	16.25	0.544
	12	23.50	13.75	19.50	15.50	0.866
	24	14.50	11.50	15.00	18.00	0.504
	52	6.00	10.00	12.50	15.25	0.263
	104	6.50	7.00	7.50	14.00	0.238
ΔPRTEE (vs week 0)	2	17.50	9.50	14.50	11.75	0.281
	4	21.50	10.25	21.50	14.50	0.758
	8	24.50	9.00	26.50	16.95	0.580
	12	27.00	13.25	29.50	16.00	0.277
	24	31.00	15.25	30.50	19.50	0.728
	52	27.00	14.50	33.50	17.88	0.583
	104	33.50	21.50	38.50	16.38	0.827

PROMs values in GG homozygotes and carriers of the A allele of the rs4930152 *VEGFB* gene polymorphism.

**PROMs**		**GG rs4930152**		**GA+AA rs4930152**		***p***
	week	median	± QD	median	± QD	**Mann-Whitney U test**
VAS	0	6.00	1.50	6.00	1.88	0.788
	2	4.00	1.50	4.00	1.50	0.815
	4	3.00	1.50	3.00	1.50	0.873
	8	2.50	1.50	3.00	2.00	0.415
	12	2.00	1.75	3.00	1.50	0.757
	24	1.00	2.50	2.00	2.00	0.349
	52	1.00	2.50	2.00	2.00	0.923
	104	1.00	2.00	1.00	1.50	0.301
ΔVAS (vs week 0)	2	2.00	1.75	1.00	1.50	0.535
	4	2.00	1.50	2.00	2.00	0.544
	8	3.00	2.50	2.00	2.00	0.424
	12	3.00	2.00	3.00	2.00	0.751
	24	3.00	2.50	2.00	1.50	0.410
	52	3.50	3.00	3.50	2.00	0.903
	104	4.00	2.50	4.00	2.50	0.336
QDASH	0	48.86	10.16	52.27	14.20	0.914
	2	40.91	13.07	38.64	17.05	0.551
	4	38.64	14.20	34.09	14.77	0.586
	8	34.09	19.89	31.82	16.48	0.806
	12	23.86	22.73	29.55	14.20	0.757
	24	14.77	21.59	25.00	20.45	0.443
	52	22.73	26.14	17.05	21.59	0.849
	104	18.18	25.00	9.09	14.78	0.117
ΔQDASH (vs week 0)	2	4.54	15.34	6.81	12.63	0.644
	4	12.50	15.33	12.49	15.91	0.890
	8	14.77	19.94	15.91	17.05	0.858
	12	18.18	16.48	18.18	18.18	0.921
	24	23.86	20.46	20.45	18.18	0.498
	52	23.86	21.59	22.72	19.32	0.734
	104	29.54	23.87	30.68	21.02	0.240
PRTEE	0	50.75	13.00	53.25	14.88	0.753
	2	28.75	12.13	31.50	18.50	0.976
	4	24.75	14.38	24.50	13.75	0.877
	8	21.25	15.25	24.25	16.88	0.668
	12	16.00	15.38	21.50	14.50	0.537
	24	9.50	17.00	18.75	16.75	0.237
	52	12.75	13.75	11.50	14.63	0.776
	104	8.50	17.50	6.50	9.75	0.218
ΔPRTEE (vs week 0)	2	17.50	13.15	14.00	13.25	0.965
	4	21.75	14.25	20.75	12.75	0.839
	8	27.75	14.25	24.50	17.50	0.961
	12	26.75	14.73	29.00	18.25	0.786
	24	33.50	17.50	30.50	19.00	0.667
	52	35.75	15.50	32.50	18.00	0.439
	104	37.00	19.75	39.00	17.25	0.085

Legend: *VEGFB*, vascular endothelial growth factor B; QD, Quartile Deviation; VAS, Visual Analog Scale; QDASH, quick version of Disabilities of the Arm, Shoulder and Hand score; PROM, Patient-Reported Outcome Measures; PRTEE, Patient-Rated Tennis Elbow Evaluation. * - statistically significant after Hochberg correction ($p = 0.007$).

Table S4. PROMs values in carriers of different genotypes of the rs594942 polymorphism of the *VEGFB* gene.

PROMs values in CC homozygotes and carriers of the T allele of the rs594942 *VEGFB* gene polymorphism.

**PROMs**		**CC rs594942**		**CT+TT rs594942**		***p***
	week	median	± QD	median	± QD	**Mann-Whitney U test**
VAS	0	6.00	2.00	6.00	1.00	0.558
	2	3.00	1.25	4.00	1.50	**0.008**
	4	3.00	1.25	4.00	2.00	0.027
	8	3.00	1.50	3.00	2.00	0.860
	12	3.00	1.50	2.00	2.00	0.653
	24	2.00	2.00	3.00	2.00	0.188
	52	1.00	1.50	2.00	2.50	0.148
	104	1.00	1.00	1.00	2.00	0.141
ΔVAS (vs week 0)	2	1.25	1.50	1.00	1.50	0.131
	4	2.00	1.50	2.00	2.00	0.465
	8	2.00	2.00	3.00	2.50	0.528
	12	2.00	1.50	3.00	2.00	0.691
	24	3.00	2.00	3.00	2.50	0.426
	52	4.00	2.00	3.00	2.50	0.373
	104	4.00	2.00	4.00	3.00	0.677
QDASH	0	52.27	17.05	52.27	12.05	0.419
	2	34.09	14.20	45.45	15.11	**0.040**
	4	31.82	12.50	40.91	15.91	0.093
	8	34.09	19.32	31.82	17.05	0.700
	12	29.55	17.05	27.27	20.45	0.806
	24	20.45	20.45	31.82	20.45	0.260
	52	13.64	23.86	27.27	23.86	0.062
	104	9.09	13.64	18.18	23.86	0.066
ΔQDASH (vs week 0)	2	6.82	13.64	6.81	12.50	0.681
	4	10.22	15.34	14.77	15.91	0.800
	8	11.36	18.19	18.18	17.05	0.121
	12	20.45	20.45	17.08	13.64	0.531
	24	21.36	18.18	20.45	19.32	0.823
	52	27.27	21.59	20.45	19.32	0.442
	104	31.81	20.45	29.55	22.73	0.588
PRTEE	0	50.50	15.25	52.50	12.50	0.543
	2	22.00	15.38	35.50	15.75	**0.039**
	4	22.00	12.00	28.50	16.75	0.106
	8	21.50	17.75	24.00	15.50	0.996
	12	21.00	14.25	19.50	16.75	0.799
	24	14.00	15.25	15.00	17.25	0.350
	52	6.00	14.00	15.00	16.00	**0.039**
	104	4.00	9.50	9.50	16.25	**0.039**
ΔPRTEE (vs week 0)	2	16.25	10.63	13.75	13.55	0.159
	4	21.75	10.88	21.00	15.25	0.585
	8	23.50	13.75	28.25	17.75	0.331
	12	28.00	17.75	29.50	15.75	0.743
	24	28.50	19.25	32.00	18.75	0.941
	52	33.50	15.75	31.50	19.00	0.213
	104	38.50	16.25	37.50	16.75	0.401

PROMs values in TT homozygotes and carriers of the C allele of the rs594942 *VEGFB* gene polymorphism.

**PROMs**	week	**TT rs594942** median	**TT rs594942** ± QD	**CT+CC rs594942** median	**CT+CC rs594942** ± QD	***p* Mann-Whitney U test**
VAS	0	6.00	1.50	6.00	1.50	0.672
	2	5.00	1.00	4.00	1.50	0.430
	4	3.00	1.00	3.00	1.50	0.723
	8	2.00	1.00	3.00	2.00	0.850
	12	3.00	2.00	2.00	1.50	0.640
	24	3.00	2.00	2.00	2.00	0.537
	52	5.00	2.50	1.00	2.00	0.070
	104	1.00	2.00	1.00	1.50	0.276
ΔVAS (vs week 0)	2	2.00	1.50	1.00	1.50	0.890
	4	3.00	1.00	2.00	2.00	0.232
	8	3.00	2.50	2.00	2.00	0.723
	12	3.00	2.00	3.00	2.00	0.741
	24	2.00	3.00	3.00	2.00	0.878
	52	1.00	2.50	4.00	2.00	0.304
	104	4.00	3.00	4.00	2.00	0.832
QDASH	0	43.18	15.77	52.27	12.50	0.282
	2	40.91	12.50	38.64	17.05	0.471
	4	36.36	14.77	36.36	14.77	0.591
	8	31.82	13.64	34.09	19.32	0.566
	12	34.09	17.05	27.27	18.18	0.511
	24	29.55	21.59	25.00	21.59	0.797
	52	34.09	14.77	15.91	22.73	0.241
	104	22.73	14.77	11.36	20.45	0.199
ΔQDASH (vs week 0)	2	2.27	12.36	6.81	13.64	0.176
	4	17.27	6.82	11.36	15.91	0.866
	8	13.63	13.63	15.91	19.32	0.770
	12	13.63	11.36	20.45	18.18	0.202
	24	15.63	5.68	22.50	20.46	0.338
	52	11.36	15.86	25.00	20.46	0.075
	104	6.82	20.01	31.82	21.02	0.086
PRTEE	0	51.50	10.25	52.50	14.25	0.952
	2	34.00	14.50	29.25	16.75	0.550
	4	29.50	11.75	24.50	14.50	0.882
	8	22.00	8.25	22.00	16.25	0.629
	12	24.00	11.25	19.50	15.00	0.440
	24	12.00	14.75	15.00	17.50	0.512
	52	20.00	13.75	11.00	14.75	0.140
	104	11.00	10.00	6.50	12.75	0.206
ΔPRTEE (vs week 0)	2	17.00	9.25	14.50	12.75	0.878
	4	21.50	9.75	21.50	13.75	0.878
	8	31.00	11.75	25.25	16.75	0.591
	12	23.50	9.00	29.00	17.50	0.565
	24	25.00	12.00	30.75	19.25	0.668
	52	17.50	14.25	33.50	18.13	0.190
	104	37.00	18.00	38.25	16.38	0.730

Legend: *VEGFB*, vascular endothelial growth factor B; QD, Quartile Deviation; VAS, Visual Analog Scale; QDASH, quick version of Disabilities of the Arm, Shoulder and Hand score; PROM, Patient-Reported Outcome Measures; PRTEE, Patient-Rated Tennis Elbow Evaluation.

Table S5. PROMs values in carriers of different genotypes of the rs595880 polymorphism of the *VEGFB* gene.

PROMs values in GG homozygotes and carriers of the T allele of the rs595880 *VEGFB* gene polymorphism.

**PROMs**		**GG rs595880**		**GT+TT rs595880**		***p***
	week	median	± QD	median	± QD	**Mann-Whitney U test**
VAS	0	6.00	2.00	6.00	1.00	0.558
	2	3.00	1.25	4.00	1.50	**0.008**
	4	3.00	1.25	4.00	2.00	0.027
	8	3.00	1.50	3.00	2.00	0.860
	12	3.00	1.50	2.00	2.00	0.653
	24	2.00	2.00	3.00	2.00	0.188
	52	1.00	1.50	2.00	2.50	0.148
	104	1.00	1.00	1.00	2.00	0.141
ΔVAS (vs week 0)	2	1.25	1.50	1.00	1.50	0.131
	4	2.00	1.50	2.00	2.00	0.465
	8	2.00	2.00	3.00	2.50	0.528
	12	2.00	1.50	3.00	2.00	0.691
	24	3.00	2.00	3.00	2.50	0.426
	52	4.00	2.00	3.00	2.50	0.373
	104	4.00	2.00	4.00	3.00	0.677
QDASH	0	52.27	17.05	52.27	12.05	0.419
	2	34.09	14.20	45.45	15.11	**0.040**
	4	31.82	12.50	40.91	15.91	0.093
	8	34.09	19.32	31.82	17.05	0.700
	12	29.55	17.05	27.27	20.45	0.806
	24	20.45	20.45	31.82	20.45	0.260
	52	13.64	23.86	27.27	23.86	0.062
	104	9.09	13.64	18.18	23.86	0.066
ΔQDASH (vs week 0)	2	6.82	13.64	6.81	12.50	0.681
	4	10.22	15.34	14.77	15.91	0.800
	8	11.36	18.19	18.18	17.05	0.121
	12	20.45	20.45	17.08	13.64	0.531
	24	21.36	18.18	20.45	19.32	0.823
	52	27.27	21.59	20.45	19.32	0.442
	104	31.81	20.45	29.55	22.73	0.588
PRTEE	0	50.50	15.25	52.50	12.50	0.543
	2	22.00	15.38	35.50	15.75	**0.039**
	4	22.00	12.00	28.50	16.75	0.106
	8	21.50	17.75	24.00	15.50	0.996
	12	21.00	14.25	19.50	16.75	0.799
	24	14.00	15.25	15.00	17.25	0.350
	52	6.00	14.00	15.00	16.00	**0.039**
	104	4.00	9.50	9.50	16.25	**0.039**
ΔPRTEE (vs week 0)	2	16.25	10.63	13.75	13.55	0.159
	4	21.75	10.88	21.00	15.25	0.585
	8	23.50	13.75	28.25	17.75	0.331
	12	28.00	17.75	29.50	15.75	0.743
	24	28.50	19.25	32.00	18.75	0.941
	52	33.50	15.75	31.50	19.00	0.213
	104	38.50	16.25	37.50	16.75	0.401

PROMs values in TT homozygotes and carriers of the G allele of the rs595880 *VEGFB* gene polymorphism.

PROMs	week	TT rs595880		GT+GG rs595880		p Mann-Whitney U test
		median	± QD	median	± QD	
VAS	0	6.00	1.50	6.00	1.50	0.672
	2	5.00	1.00	4.00	1.50	0.430
	4	3.00	1.00	3.00	1.50	0.723
	8	2.00	1.00	3.00	2.00	0.850
	12	3.00	2.00	2.00	1.50	0.640
	24	3.00	2.00	2.00	2.00	0.537
	52	5.00	2.50	1.00	2.00	0.070
	104	1.00	2.00	1.00	1.50	0.276
ΔVAS (vs week 0)	2	2.00	1.50	1.00	1.50	0.890
	4	3.00	1.00	2.00	2.00	0.232
	8	3.00	2.50	2.00	2.00	0.723
	12	3.00	2.00	3.00	2.00	0.741
	24	2.00	3.00	3.00	2.00	0.878
	52	1.00	2.50	4.00	2.00	0.304
	104	4.00	3.00	4.00	2.00	0.832
QDASH	0	43.18	15.77	52.27	12.50	0.282
	2	40.91	12.50	38.64	17.05	0.471
	4	36.36	14.77	36.36	14.77	0.591
	8	31.82	13.64	34.09	19.32	0.566
	12	34.09	17.05	27.27	18.18	0.511
	24	29.55	21.59	25.00	21.59	0.797
	52	34.09	14.77	15.91	22.73	0.241
	104	22.73	14.77	11.36	20.45	0.199
ΔQDASH (vs week 0)	2	2.27	12.36	6.81	13.64	0.176
	4	17.27	6.82	11.36	15.91	0.866
	8	13.63	13.63	15.91	19.32	0.770
	12	13.63	11.36	20.45	18.18	0.202
	24	15.63	5.68	22.50	20.46	0.338
	52	11.36	15.86	25.00	20.46	0.075
	104	6.82	20.01	31.82	21.02	0.086
PRTEE	0	51.50	10.25	52.50	14.25	0.952
	2	34.00	14.50	29.25	16.75	0.550
	4	29.50	11.75	24.50	14.50	0.882
	8	22.00	8.25	22.00	16.25	0.629
	12	24.00	11.25	19.50	15.00	0.440
	24	12.00	14.75	15.00	17.50	0.512
	52	20.00	13.75	11.00	14.75	0.140
	104	11.00	10.00	6.50	12.75	0.206
ΔPRTEE (vs week 0)	2	17.00	9.25	14.50	12.75	0.878
	4	21.50	9.75	21.50	13.75	0.878
	8	31.00	11.75	25.25	16.75	0.591
	12	23.50	9.00	29.00	17.50	0.565
	24	25.00	12.00	30.75	19.25	0.668
	52	17.50	14.25	33.50	18.13	0.190
	104	37.00	18.00	38.25	16.38	0.730

Legend: *VEGFB*, vascular endothelial growth factor B; QD, Quartile Deviation; VAS, Visual Analog Scale; QDASH, quick version of Disabilities of the Arm, Shoulder and Hand score; PROM, Patient-Reported Outcome Measures; PRTEE, Patient-Rated Tennis Elbow Evaluation.

Table S6. PROMs values for different genotypes of the *VEGFB* gene polymorphisms in the additive model.

PROM	week	Genotype of rs72922019 CC		CT		TT		*p* value			
		Median	±QD	Median	±QD	Median	±QD	Kruskal-Wallis test	CC vs CT	TT vs CT	CC vs TT
VAS	2	4.00	1.50	4.00	1.50	2.00	1.50	0.011*	1.000	**0.009***	**0.049**
QDASH	2	40.91	13.07	43.18	17.05	23.86	14.77	0.023	1.000	**0.023**	**0.047**
PRTEE	2	28.75	12.13	36.50	16.75	17.50	9.00	0.027	1.000	**0.021**	0.130

PROM	week	Genotype of rs12366035 CC		CT		TT		*p* value			
		Median	±QD	Median	±QD	Median	±QD	Kruskal-Wallis test	CC vs CT	TT vs CT	CC vs TT
VAS	2	4.00	1.50	4.00	1.50	2.00	1.50	0.005*	1.000	**0.004***	**0.024**
QDASH	2	40.91	13.07	43.18	17.05	23.86	14.77	0.013	1.000	**0.012**	**0.026**
PRTEE	2	28.75	12.13	36.50	16.75	17.50	9.00	0.021	1.000	**0.016**	0.098

PROM	week	Genotype of rs4930152 GG		GA		AA		*p* value			
		Median	±QD	Median	±QD	Median	±QD	Kruskal-Wallis test	GG vs GA	AA vs GA	GG vs AA
VAS	2	4.00	1.50	4.00	1.50	2.00	1.50	0.005*	1.000	**0.003***	**0.024**
QDASH	2	40.91	13.07	43.18	17.05	23.86	14.77	0.012	1.000	**0.011**	**0.026**
PRTEE	2	28.75	12.13	36.50	16.75	17.50	9.00	0.019	0.996	**0.015**	0.095

PROM	week	Genotype of rs594942 CC		CT		TT		*p* value			
		Median	±QD	Median	±QD	Median	±QD	Kruskal-Wallis test	CC vs CT	TT vs CT	CC vs TT
VAS	2	3.00	1.25	4.00	1.50	5.00	1.00	0.028	**0.035**	1.000	0.425
	4	3.00	1.25	4.00	2.00	3.00	1.00	0.043	**0.042**	0.762	1.000

PROM	week	Genotype of rs595880 GG		GT		TT		*p* value			
		Median	±QD	Median	±QD	Median	±QD	Kruskal-Wallis test	GG vs GT	TT vs GT	GG vs TT
VAS	2	3.00	1.25	4.00	1.50	5.00	1.00	0.028	**0.035**	1.000	0.425
	4	3.00	1.25	4.00	2.00	3.00	1.00	0.043	**0.042**	0.762	1.000

Legend: *VEGFB*, vascular endothelial growth factor B; QD, Quartile Deviation; PROMs, patient-reported outcome measures; VAS, Visual Analog Scale; QDASH, quick version of Disabilities of the Arm, Shoulder and Hand score; PRTEE, Patient-Rated Tennis Elbow Evaluation. * - statistically significant after Hochberg correction ($p = 0.012$).

Table S7. Median (±QD) of blood morphological parameters for genotypes of the *VEGFB* gene polymorphisms in the additive model.

PROM	Genotype of rs72922019						*p* value			
	CC		CT		TT					
	**Median**	**±QD**	**Median**	**±QD**	**Median**	**±QD**	**Kruskal-Wallis test**	**CC vs CT**	**TT vs CT**	**CC vs TT**
EOS [10^{*9}/L]	0.21	0.09	0.12	0.04	0.13	0.05	0.025	**0.026**	1.000	0.313

PROM	Genotype of rs12366035						*p* value			
	CC		CT		TT					
	**Median**	**±QD**	**Median**	**±QD**	**Median**	**±QD**	**Kruskal-Wallis test**	**CC vs CT**	**TT vs CT**	**CC vs TT**
EOS [10^{*9}/L]	0.21	0.09	0.12	0.04	0.13	0.05	0.025	**0.026**	1.000	0.313

PROM	Genotype of rs4930152						*p* value			
	GG		GA		AA					
	**Median**	**±QD**	**Median**	**±QD**	**Median**	**±QD**	**Kruskal-Wallis test**	**CC vs CT**	**TT vs CT**	**CC vs TT**
EOS [10^{*9}/L]	0.21	0.09	0.12	0.04	0.13	0.05	0.025	**0.026**	1.000	0.313

PROM	Genotype of rs594942						*p* value			
	CC		CT		TT					
	**Median**	**±QD**	**Median**	**±QD**	**Median**	**±QD**	**Kruskal-Wallis test**	**CC vs CT**	**TT vs CT**	**CC vs TT**
RDW [%]	12,00	0,30	11,80	0,35	12,40	0,40	0,001*	**0,018**	**0,005**	0,345
EOS [10^{*9}/L]	0,13	0,04	0,19	0,10	0,19	0,06	0,024	**0,021**	1,000	0,813
MONO [%]	5,50	1,50	4,50	1,40	4,50	0,30	0,032	**0,041**	1,000	0,375

PROM	Genotype of rs595880						*p* value			
	GG		GT		TT					
	**Median**	**±QD**	**Median**	**±QD**	**Median**	**±QD**	**Kruskal-Wallis test**	**CC vs CT**	**TT vs CT**	**CC vs TT**
RDW [%]	12,00	0,30	11,80	0,35	12,40	0,40	0,001*	**0,018**	**0,005**	0,345
EOS [10^{*9}/L]	0,13	0,04	0,19	0,10	0,19	0,06	0,024	**0,021**	1,000	0,813
MONO [%]	5,50	1,50	4,50	1,40	4,50	0,30	0,032	**0,041**	1,000	0,375

Legend: *VEGFB*, vascular endothelial growth factor B; QD, Quartile Deviation; EOS, eosinophils; RDW, red blood cell distribution width; MONO, monocytes. * - statistically significant after Hochberg correction ($p = 0.001$).

Table S8. The distribution of genotype frequencies of the *VEGFB* gene's polymorphisms in patients with different comorbidities.

Model of Heredity	Comorbidity	SNP	Genotype	Presence of Comorbidity			No Comorbidity			*p* value
				n	genotype carriers [%]	patients with comorbidities [%]	n	genotype carriers [%]	patients without comorbidities [%]	
Additive	autoimmune diseases	rs72922019	CC	12	21.43	48.00	44	78.57	41.12	0.021
			CT	6	10.34	24.00	52	89.66	48.60	
			TT	7	38.89	28.00	11	61.11	10.28	
		rs12366035	CC	12	21.43	48.00	44	78.57	41.51	0.015
			CT	6	10.34	24.00	52	89.66	49.06	
			TT	7	41.18	28.00	10	58.82	9.43	
		rs4930152	GG	12	21.43	48.00	44	78.57	41.12	0.013
			AG	6	10.17	24.00	53	89.83	49.53	
			AA	7	41.18	28.00	10	58.82	9.35	
	heart failure	rs594942	CC	1	1.59	25.00	62	98.41	49.21	0.00005*
			CT	0	0.00	0.00	54	100.00	42.86	
			TT	3	23.08	75.00	10	76.92	7.94	
		rs595880	GG	1	1.59	25.00	62	98.41	49.21	0.00005*
			TG	0	0.00	0.00	54	100.00	42.86	
			TT	3	23.08	75.00	10	76.92	7.94	
Dominant/recessive	hypertension	rs72922019	CC	12	21.43	66.67	44	78.57	38.60	0.047
			CT+TT	6	7.89	33.33	70	92.11	61.40	
		rs4930152	GG	12	21.43	66.67	44	78.57	38.60	0.047
			AG+AA	6	7.89	33.33	70	92.11	61.40	
	autoimmune diseases	rs72922019	TT	7	38.89	28.00	11	61.11	10.28	0.045
			CT+CC	18	15.79	72.00	96	84.21	89.72	
		rs12366035	TT	7	41.18	28.00	10	58.82	9.43	0.031
			CT+CC	18	15.79	72.00	96	84.21	90.57	
		rs4930152	AA	7	41.18	28.00	10	58.82	9.35	0.030
			AG+GG	18	15.65	72.00	97	84.35	90.65	
	heart failure	rs594942	TT	3	23.08	75.00	10	76.92	7.94	0.00038*
			CT+CC	1	0.85	25.00	116	99.15	92.06	
		rs595880	TT	3	23.08	75.00	10	76.92	7.94	0.00038*
			TG+GG	1	0.85	25.00	116	99.15	92.06	

Legend: *VEGFB*, vascular endothelial growth factor B, SNP, single nucleotide polymorphism. * - statistically significant after Hochberg correction ($p = 0.00038$).
